# A Treatise on the Medullary Fungus of the Testicle

**Published:** 1836-04

**Authors:** 


### Art VI.

*Ueber den Markschwamm der Hoden.* Vom Dr. Otto Baring, practischem Arzte und Wundarzte in Hannover. Göttingen, 1833. 8vo. pp. 237.

*A Treatise on the Medullary Fungus of the Testicle.* By Otto Baring, Doctor of Medicine and Surgery.

Patient industry, indefatigable research, and voluminous results have long characterized the labours of the German writers, and the author of this work has omitted nothing which could tend to establish his nationality. We have here above two hundred octavo pages, devoted to the description of one particular disease in one particular organ of the body: and yet every page bears the stamp of much patient investigation and careful diligence bestowed upon the composition before it was committed to the press. Dr. Baring has dived deeply into the writings of his predecessors and his contemporaries, and, with one or two slight exceptions, we believe there is not an author who has published on the subject of fungus during the last five and thirty years whose opinions are not collected and arranged in the work before us. Not only have the well known treatises on this subject by English, French, German, and Italian surgeons been analyzed and compared, but monographs, papers, and single cases have been hunted out from the different journals in which they first saw this light, and where they might have slumbered for ever, had they not been discovered and seized upon by this indefatigable inquisitor.

It is much to be regretted, that many of the German authors, who raise such vast monuments of their industry and patience, never arrive at what should be the most useful results of their labour and ingenuity; that, notwithstanding the mass of information which they love to collect, embracing every thing which is, or ever has been known on the subject, they do not always avail themselves of the opportunity which such an intimate knowledge, so much accumulated evidence must afford them, of giving to the world those rational and sound deductions, and inferences from the experience of our predecessors, which alone can render that experience beneficial to others. It is only the zealous collector of scattered observations, and the judicious collator of discordant opinions, who can be considered as properly fitted to weigh, and pronounce judgment on their accuracy and importance. No second person can thoroughly enter into the author's ideas; and the conclusions arrived at by him who has carefully waded through the mass of evidence, must necessarily be sounder, and more valuable than those of the mere reader, who finds the detail ready furnished for his hasty perusal. The learned and industrious author of the present treatise is not entirely free from the charge implied in these remarks; still it will be found that his labours are far from being unproductive of important practical fruits.

The first two chapters of Dr. Baring's work contain a literary, historical, and chronological account of the fungous disease in general, as it shows itself in the various tissues of the body. In these the author has collected and compared the different notices which have been furnished from time to time by the English, French, German, and Italian surgeons, respecting this malady; and with considerable ability has succeeded in clearing away the obscurity which enveloped it, and reconciling the discrepancies, which in great measure were rather apparent than real, and resulted chiefly from the endless variety of names adopted by different writers to signify one and the same affection of the body.

It is flattering to our national vanity to be assured by so competent an authority, that the English medical writers were the first to point out and describe clearly the fungous disease as distinguished from other malignant disorders.

The French surgeons seem to have entirely misapprehended the nature of the disease as described by Hey and Burns, probably owing to the somewhat inappropriate name of fungus-hæmatodes given to it by those authors. To this name they attached the idea of varicose tumours, nævi, aneurisms by anastomosis, and in fact every thing but that of the disease which was intended to be described; and whilst they identified the fungus-hæmatodes with a morbid and dilated condition of the blood-vessels, they naturally missed the true character of the disease which the English had designated by the above appellation. Thus it happened, that Roux, in the year 1814, on being shown a case of fungous testicle, which was extirpated in his presence by Sir Astley Cooper at Guy's Hospital, expressed his astonishment, and confessed that he as well as his countrymen had entirely mistaken the descriptions of the English authors on this subject: at the same time stating that the disease in question was that variety of scirrhus which the French had long known under the name of soft cancer, remarkable for its rapid progress, soft and pulpy texture, &c. Indeed, most of the French authors, including Dupuytren, Delpech, Cruveilhier, and Breschet, who have accurately described this malady under a variety of names, most of which have reference to its cerebriform structure, insist upon its being a variety of scirrhus, frequently forming the last stage or termination of true cancerous affections. It is right however to observe that Laennec appears to have distinguished the disease, as early as 1805, by the name of Dégénérescence Cérébriforme, and subsequently, in his article on Pathological Anatomy in the Dict. des Sc. Méd., he gives a masterly description of it under the name of "Matière Encéphaloide ou Cérébriforme."

Among the Italian surgeons, Scarpa appears to have devoted considerable attention to this disease; but maintains that it only occurs in the cellular tissue under the skin, and the interstices of the muscles, and, somewhat unaccountably, denies its existence as attacking the substance of the different viscera. The medullary fungus of the testicle and the mamma, he seems to consider as a strumous affection of those organs. It is unnecessary to say how much he erred in this opinion.

It may readily be imagined that amongst the German writers, there were more than one who endeavoured to reconcile the discrepancies of other medical historians, and to form some more distinct arrangement of those diseases which were known under the generic cognomen of malignant. Accordingly we find that Meckel, Casper, Langenbeck, Walther, Otto, and many others have contributed largely on this subject. They appear by common consent to have given the name of medullary fungus to the disease in question, and the result of their researches has an evident tendency to distinguish it from cancer, although it must be allowed that the two morbid affections become occasionally combined in the same individual. There exists a remarkable coincidence, in many points, between these authors and the English writers on the same subject. Dr. Baring has adopted the term medullary fungus as being most characteristic of the nature of the disease, and generally used by his countrymen.

After the literary and historical account of the medullary fungus as a distinct disease, the author enters upon a general description of it, noticing its origin, development, and termination, as it occurs in the different tissues and regions of the human body. As, however, he does not pretend to have made any discoveries with respect to the anatomical texture of the disease, we must pass over this part of the work, valuable as it is, as a compendium of all that is known on the subject.

Having at length arrived at the proper subject of his book, the medullary fungus of the testicle, the author presents us with a very full, clear, and satisfactory account of the disease, from its commencement as indicated by a slight enlargement and hardness of the organ, to its termination in the death of the patient, if not previously arrested by the operation of extirpation. We shall forbear to make any extracts, as the history corresponds in a remarkable degree, not only as regards the leading points, but in most of the minuter particulars, with the description given of the fungoid disease by Sir A. Cooper, in his splendid work on the testis. Indeed it throws if possible additional value on the evidence of our distinguished countryman, and confirms the accuracy of his observation, when we find the same conclusions are arrived at by such different methods. Sir Astley has given us a vivid and graphic account of this disease from his own long and most extensive experience. Dr. Baring has added to his own personal observations a patient investigation of the same disease, as detailed by the most eminent surgeons of all countries. He has weighed the force of their evidence, tested their accuracy by comparing them against each other, and by a judicious selection of facts has arrived at conclusions differing but slightly from those of his illustrious predecessors.

Sir Astley Cooper's experience has taught him that the fungoid disease always commences in the body of the testis. From the researches of Dr. Baring we learn, that the epidydimis is occasionally the primary seat of the affection. The personal observation of Sir A. Cooper, and the collected experience of Dr. Baring are also at variance, respecting the ulceration of the scrotum in the latter stages of the disease, and the formation of granulations bearing the true medullary fungoid character. Sir Astley appears to consider this as not a very unfrequent termination of the complaint, while our author arms himself with the authority of a host of continental surgeons, and decides that it is of extremely rare occurrence. He seems to consider the tardy ulceration, or rather the non-ulceration of the scrotum as a phenomenon, for which he confesses that he is utterly unable to account, and expends much labour and considerable ingenuity in endeavouring to prove that there is a disinclination for the disease to reach the surface, which is peculiar to the fungoid affection of the testicle.

It has indeed always struck us, that in portraying the termination of this disease in its worst and most loathsome form, Sir A. Cooper has unintentionally given the reader to understand that such a termination is more frequent than common experience would induce us to suppose, and he subsequently remarks, that the patient will often sink under symptoms of general cachexia before the skin has given way. There can be no doubt, that medullary fungus of the testicle has not the same tendency to produce ulceration of the skin, as a similar affection of any other part of the body, equally superficial in situation, and this peculiarity, which is so puzzling to Dr. Baring, admits we conceive of an easy explanation on rational grounds. The extreme capability of extension which the scrotum possesses, as evinced in hydrocele, and more especially in large irreducible herniæ, readily allows the testicle to acquire an enormous size, before any great degree of tension or discoloration of the skin takes place: the disease thus becomes fully developed, not only in the original seat, but frequently in other more vital organs of the body, and the patient sinks from constitutional symptoms, while the only visible local affection is yet in progress towards what we are led to consider the climax of the disorder. We may also add, that, owing to the free communication between the testicle and the belly, the disease is rapidly extended along the absorbent vessels of the cord; and the entire lymphatic system of the abdominal cavity may become extensively affected, long before there is any tendency to ulceration on the surface of the scrotum.

We must be allowed to express a doubt as to the correctness of Dr. Baring's opinions respecting the duration of the fungous disease, which, he says, is frequently several years: fourteen years is his maximum. We are well aware that the complaint often supervenes upon chronic enlargement of the testicle, injuries of the organ, or other exciting causes, but our own experience has taught us that when the true fungoid character is once established, its progress is much more rapid than is here described, generally terminating the life of the patient within a twelvemonth from the commencement.

Dr. Baring also refers to the secondary enlargement of the inguinal glands as a usual accompaniment of fungoid testicle, and places it among the early symptoms; at the same time saying that the period of its appearance is very uncertain, as it sometimes comes on at the commencement, sometimes only at the latter stages of the disease. This, according to our experience, is hardly correct. The inguinal glands do not commonly participate in the complaint; and never until the skin of the scrotum, or the cellular membrane immediately subjacent, has become affected. The lumbar glands within the abdomen take on the morbid action much more readily, because they receive the absorbents direct from the testicle; whereas, the inguinal glands which communicate only with the scrotum, do not sympathise until ulceration is about to take place.

Our limits will not allow us to follow Dr. Baring through the long train of constitutional symptoms which characterize the progress of this disease, and the miserable state of cachexia which at length terminates the life of the sufferer; neither can we give even a mere outline of the vast number of cases, accompanied by post-mortem examinations, which are brought forward to illustrate the pathology of medullary fungus, as first developed in the testicle, and subsequently extending to nearly all the viscera and tissues of the body. These are extended through a space of more than sixty pages, and contain the history of dissections made by all the most eminent surgeons and pathologists of Europe, including some interesting cases in which fungoid tubercles had become developed on the surface of the arachnoid tunic and within the substance of the brain. The following are the author's conclusions respecting the nature and mode of propagation of the fungoid disease from its original seat in the testicle to the other textures of the body:—

"1. It is not the primary fungoid disease of the testicle itself, but the subsequent degeneration taking place in the abdominal and thoracic cavities, which terminates the life of the patient.

"2. These abdominal and thoracic degenerations are either fungoid or closely allied to fungus in all their physical qualities.

"3. A careful dissection will enable us to demonstrate the progress of the fungoid disease, step by step, as it becomes extended (through the absorbents of the cord) from the testicle to the more distant organs of the body.

"4. Although the medullary fungus is found to attack almost every organ as a primary disease, yet in the cases above alluded to, it must be regarded, as well in the larger parenchymatous viscera as in the lymphatic system itself, as a secondary and consecutive, and not as a primary affection." (P. 108.)

These views, as the author justly remarks, involve a most important principle as regards the prognosis to be given in incipient cases of fungoid testicle, and the hopes which may be afforded by an early extirpation of the original seat of the disease.

The fourth chapter, which contains the diagnosis of fungoid testicle, constitutes, in our opinion, the most useful portion of Dr. Baring's work. In this the author gives us a minute, faithful, and perspicuous comparison, between the disease in question and every other affection of the testicle with which it can possibly be confounded. The symptoms that distinguish fungous from scirrhous disease are thus arranged:

"(*a*). True scirrhus of the testicle is a very rare complaint in advanced age: fungus, on the other hand, is by no means uncommon; but more frequently occurs at a much earlier period of life.

"(*b*). Scirrhus is much less rapid in its progress than fungus. Meckel judiciously observes that fungus destroys life in as many months as cancer requires years. At the same time it cannot be denied that occasionally fungus is also tardy in its course.*

"(*c*). The external form of the testicle differs in each affection. The cartilaginous stony hardness; the uneven tuberculated surface, conveying a sense of solidity but not the slightest elasticity to the touch; the irregularity of shape assumed as the oval figure becomes lost; all of which characterize scirrhus from its commencement, are wanting in fungus. This latter is soft and elastic to the touch, conveys a deceptive feeling of fluctuation, and always attains its natural oval form. Scirrhus never reaches the size which medullary fungus attains in the last stage of its development.

"(*d.*) In scirrhus, the cord presents the same knotty hardness as the testicle, so that it often enlarges to more than three times its natural volume. This is not the case in fungus, where the cord seldom or never undergoes any very important change; it never becomes very hard, but on the contrary is yielding and elastic to the touch; and this condition, as we have mentioned above, is produced merely by the distension and varicose state of its lymphatic vessels.†

"(*e.*) When scirrhus is allowed to run its course, the testicle almost invariably contracts adhesions to the dartos, followed by ulceration of the skin and the protrusion of fungoid excrescences. This seldom or never takes place in the medullary fungus.

"(*f.*) The absorbent glands which become enlarged during the progress of scirrhous disease never attain that size or present such frightful masses as we find supervening upon the fungoid affection.

"The anatomical structure of the two morbid affections is no less different. The scirrhous testicle presents a hard white compact mass, resembling bacon, and intersected with fibres of a cartilaginous consistence: it feels like gristle, and is of a laminated texture furnished with divergent white striæ. Fungoid testicle, on the other hand, is characterized by the softness and sponginess of its texture, the varying nature of which has already been described.

* We have already had occasion to notice what we considered an erroneous opinion of Dr. Baring respecting the occasional slow progress of the fungous disease.

† Sir Astley Cooper entertains a different opinion, as he says the cord frequently presents an immense enlargement, accompanied with induration.

"There is one circumstance which might occasionally mislead us in our investigation. It sometimes happens that during the development of medullary fungus in its advanced stage, particularly during the softening down of the encephaloid structure, isolated hard spots become apparent to the touch. These, however, are by no means essential to the fungoid structure, but depend upon small osseous or cartilaginous deposits, which now and then would seem to be accidentally produced in the last stage of this disease of the testicle. Scirrhus is, from the commencement, hard over the whole surface of the tumour, while the soft spongy nature of the swelling forms the chief characteristic of fungus." (P. 113.)

We may here observe that the fungoid testicle in its incipient stage presents an almost scirrhous hardness, so as to render a correct diagnosis at this period extremely difficult. A section of the part will however shew, that this solidity of texture is more apparent than real, and depends mainly on the unyielding nature of the tunica albuginea, which for a time masks the character of the softening mass contained within it. It is not until this tunic gives way under the pressure, that the elastic pulpy structure of the disease becomes clearly developed to the touch.

But it is the differences which distinguish the fungous from the non-malignant diseases of the testicle, that are of the highest importance to the surgeon, since a mistaken view may, in the one case, involve the life of the patient, which might have been saved, or at any rate prolonged, by an early operation: or, on the other hand, may occasion the loss of an important organ where no necessity existed for its removal.

It is not the broad and well marked symptoms which accompany the full development of disease, but the more delicate and minuter shades of difference characterizing the first appearance of deviation from the normal structure, which should be carefully studied by the surgeon, to enable him to draw a distinction between the malignant affections and those of a milder character. His opinion will be of little value unless it be given before the cachectic state and failing powers of the patient, and the extension of the disease to the lymphatic system, have stamped in too fatal characters the nature of the malady. How often does it happen that the opinion of the surgeon becomes decided only by those symptoms, which at the same time declare that the period is already gone by when the antidote might have been applied!

The pages devoted by Dr. Baring to this subject, in which he treats of the diagnosis of fungus from hydrocele, hydatids, chronic enlargement, and other non-malignant affections, will not admit of condensation, but contain much valuable information carefully selected and judiciously arranged. The author has hardly laid sufficient stress on the general aspect of the patient, as a valuable portion of our diagnosis, in the early stage of the disease. Neither should the use of mercury be lost sight of as a means of determining the nature of the swelling. Sir Astley Cooper strongly advises that in all doubtful cases the patient should be put fairly under its influence. If the enlargement be of a non-malignant character, such a plan, combined with local treatment, will rarely fail of producing some impression; if, on the other hand, we find that instead of affording relief, the symptoms become aggravated under the medicine employed, then our worst suspicions will become confirmed.

Amongst the many diseases enumerated by Dr. Baring as liable to be mistaken for fungus, he has omitted to mention hæmatocele; which is sometimes produced in so insidious a manner as to render its character a matter of considerable doubt, even to the most experienced judges. It is true that in the majority of cases of hæmatocele, the nature of the disease is rendered manifest by the history of its formation: the receipt of a severe blow on the part, and the swelling rapidly succeeding to the injury, will generally afford sufficient evidence that blood has become extravasated into the cavity of the tunica vaginalis. But it sometimes happens that the symptoms of this complaint are particularly obscure, and that the effusion occurs without any previous injury. Sir Astley Cooper has recorded a case where the testicle was removed in a perfectly healthy state, together with a large quantity of coagulated blood, which filled up the cavity of the tunica vaginalis and simulated the appearance of medullary fungus. Two instances have come under our observation, in which the symptoms were so nicely balanced, that the patient was placed on the operating table in order that an explorative incision might be made into the scrotum, to be followed by the removal of the testicle, or the coagulum, according as the case should turn out to be fungus or hæmatocele: in the one, the incision exposed a mass of coagulated blood; in the other, a true malignant enlargement of the gland itself. The cachectic habit of body and the general want of tone, which would be likely to produce, or at any rate to accompany, the spontaneous effusion of blood, tends to throw additional obscurity over the distinguishing symptoms of these two diseases, and whether a hæmatocele be mistaken for fungus, or fungus for hæmatocele, the result may be equally disastrous to the welfare of the patient and to the reputation of the surgeon.

The fifth chapter contains Dr. Baring's observations on the etiology of the fungous disease, or the causes which are supposed to produce it; and he freely confesses his inability to explain away the obscurity in which this subject is involved. The proximate cause may frequently be traced to a blow or squeeze, or some mechanical injury offered to the part, which excites an inflammation more or less acute, or very often of an inappreciable character, but producing a gradual change in the structure and organization of the testicle.

Although such a consequence resulting from a simple injury, clearly evinces a state of system favorable to the formation of malignant disease, Dr. Baring deprecates the opinion that the appearance of fungus in one particular organ (whether supervening after violence or arising spontaneously) is to be considered as the indication, or rather as the decided proof, of a fungoid tendency pervading the whole body. He contends that the subsequent degeneration of structure which takes place in the various viscera of the body, is, in the majority of cases, the direct consequence and result of the primary affection of the testicle; and brings forward the appearance presented by post-mortem examinations, to prove that a chain of morbid communication will be found to exist between that organ and the internal structures which subsequently become diseased. The testicle he considers as the starting-point, and the lymphatic system the vehicle by which the fungous degeneration is propagated, as he affirms, by gradual, successive, and regular steps; first to the neighbouring organs, and lastly to the more remote tissues.

We fear that the favorable opinions which Dr. Baring entertains respecting the local nature of the disease, are more calculated to encourage the hopes and allay the apprehension of patients, than to relieve medical readers from the apprehension of the existence of a fungous diathesis. We think it would not be difficult to prove that in very many cases, where an operation had been performed, the disease has been found to return in situations which had no apparent connexion with the original seat of the malady. The error, however, if error it be, is certainly on the safe side, as it will have the effect of inducing us to watch the first appearance of the complaint, carefully to study the diagnostic symptoms in its incipient stage, and to give the patient the only chance that can be afforded him, by an early removal of the diseased organ.

In respect to the prognosis to be given in this disease, it is hardly necessary to say that Dr. Baring anticipates a more favorable and permanently successful result from an early operation, than the majority of his professional brethren would feel themselves justified in entertaining. But it is right to mention that he corroborates his opinion by the history of four cases, in which the operation of castration was performed by Rust of Berlin, by Langenbeck of Göttingen, and by Hagedorn of Stade. In two of these cases, a period of five years, in another of three years, and in the fourth of two years, had elapsed since the removal of the testicle; and the patients were still in the enjoyment of perfect health, and had not experienced the slightest return of the complaint.

We shall conclude our review of Dr. Baring's work by quoting, in his own words, certain rules laid down, by which our opinion is to be guided, when called upon to decide on the propriety of performing an operation; and by which we may in some measure estimate the prospect of permanent relief afforded to our patient by the removal of the testicle.

"The operation of castration is contraindicated in those cases where both testicles have become affected at the same time, or where the disease of these organs is either preceded or accompanied, or becomes shortly followed by other swellings of a similar nature on the different parts of the body; more especially when internal tuberculous enlargements can be felt through the abdominal parietes. Any existing derangement of the respiratory and digestive functions should always excite our suspicion, more particularly if these untoward symptoms refuse to yield under the exhibition of appropriate internal remedies. In all such cases, we shall feel ourselves not only authorized, but compelled to declare our opinion, that the local disease of the testicle is indeed the symptom of a disposition to fungoid degeneration pervading the whole system; of a state which no operation can remedy. Again, the spontaneous occurrence of fungus in the testicle, unpreceded by any mechanical injury or lesion of the part, must always be regarded with a suspicious eye; as it indicates a constitutional tendency to the disease, and requires a more than usually careful investigation into the general health and condition of the patient before an operation is decided upon.

"It is worthy of remark, that although the secondary enlargement of the inguinal glands, supervening upon fungoid affections of the testicle, undoubtedly indicates the absorption of the poison; yet, such enlargement affords by no means so unfavorable a prognosis as those appearances which evince the extension of the disease through the agency of the spermatic cord: in the former case the absorption has taken place by means of the lymphatics of the scrotum only, and, under such circumstances, should the cord still remain healthy, should the pelvis and belly be still free from disease, the chance of an operation is not altogether to be rejected; although it presents a much less encouraging prospect than might be entertained if the glands of the groin still preserved their natural condition.

"On the other hand, we may consider that the operation of castration should be had recourse to in all those cases where the ordinary health of the patient remains undisturbed during the development of the disease in the testicle, and especially where the fungoid enlargement appears to result from some external cause or mechanical injury: in short, wherever, after a careful investigation into the constitutional symptoms, we feel justified in considering the disease as a local affection, unaccompanied by any of those contraindications which have already been detailed."—(P. 195.)

While prosecuting our analysis of Dr. Baring's work, we have occasionally compared his voluminous details with the concise and graphic descriptions of Sir Astley Cooper; and while we eagerly render to our German brother all the praise which he has so justly earned by his indefatigable research and industry; wecannot withhold the expression of our gratitude towards our own illustrious countryman for having compressed the result of so much experience into so small a space. Dr. Baring has furnished us with a book of reference, in which the enquiring student will find all that has ever been written or is known on the subject; Sir Astley Cooper has presented us with a practical manual, invaluable to the surgeon from its conciseness and accuracy. Drawing a comparison between the two authors, we may indeed say, that, to the one, little could be added to render it more useful; from the other nothing could be taken away without detracting from its worth.

